# A Rationally Designed Hsp70 Variant Rescues the Aggregation-Associated Toxicity of Human IAPP in Cultured Pancreatic Islet β-Cells

**DOI:** 10.3390/ijms19051443

**Published:** 2018-05-12

**Authors:** Marie Nicole Bongiovanni, Francesco Antonio Aprile, Pietro Sormanni, Michele Vendruscolo

**Affiliations:** Centre for Misfolding Diseases, Department of Chemistry, University of Cambridge, Cambridge CB2 1EW, UK; mb987@cam.ac.uk (M.N.B.); ps589@cam.ac.uk (P.S.)

**Keywords:** rational design, molecular chaperones, Hsp70, protein aggregation, hIAPP, amylin

## Abstract

Molecular chaperones are key components of the protein homeostasis system against protein misfolding and aggregation. It has been recently shown that these molecules can be rationally modified to have an enhanced activity against specific amyloidogenic substrates. The resulting molecular chaperone variants can be effective inhibitors of protein aggregation in vitro, thus suggesting that they may provide novel opportunities in biomedical and biotechnological applications. Before such opportunities can be exploited, however, their effects on cell viability should be better characterised. Here, we employ a rational design method to specifically enhance the activity of the 70-kDa heat shock protein (Hsp70) against the aggregation of the human islet amyloid polypeptide (hIAPP, also known as amylin). We then show that the Hsp70 variant that we designed (grafted heat shock protein 70 kDa-human islet amyloid polypeptide, GHsp70-hIAPP) is significantly more effective than the wild type in recovering the viability of cultured pancreatic islet β-cells RIN-m5F upon hIAPP aggregation. These results indicate that a full recovery of the toxic effects of hIAPP aggregates on cultured pancreatic cells can be achieved by increasing the specificity and activity of Hsp70 towards hIAPP, thus providing evidence that the strategy presented here provides a possible route for rationally tailoring molecular chaperones for enhancing their effects in a target-dependent manner.

## 1. Introduction

Molecular chaperones help maintain protein homeostasis by transiently binding their protein substrates to assist them in folding and trafficking [1,2], and to prevent them from aggregating and exerting cytotoxic effects [2,3,4,5,6,7,8]. It has thus been suggested that these molecules could be used as potential agents against protein misfolding diseases [9,10,11,12,13]. Such diseases arise from the misfolding, and aberrant deposition of otherwise normally soluble proteins, leading to a loss of function and a gain of toxicity [3,4,14,15,16,17,18,19,20,21,22,23]. A significant advantage of molecular chaperones over other protein aggregation inhibitors, such as small molecules and antibodies, arises from their potent activity at substoichiometic concentrations [24,25,26,27]. In order to fully exploit these opportunities, however, strategies to increase the specificity of the interactions of molecular chaperones with their target substrates should be developed. This aspect is important as, typically, molecular chaperones have multiple substrates, and, therefore, altering them in a non-specific manner may lead to a variety of potentially harmful off-target effects.

Among molecular chaperones, heat shock protein 70 kDa (Hsp70) has been the focus of much interest for protein misfolding diseases since it plays essential roles in protein homeostasis in physiological and stress conditions [5,28,29], it is associated with protein deposits [5,10], and it has been shown to reduce aggregate cytotoxicity in mammalian systems [30,31,32,33]. In biomedical research, the strategy of using Hsp70 to mediate toxic protein aggregation has primarily been based so far on increasing its stoichiometric ratio to proteins [34,35,36,37,38] or on stimulating its activity [6,36]. Attempts to alter the activity of Hsp70 by targeting its nucleotide-binding domain (NBD) have also been made [39,40]. These approaches may, however, lead to non-specific substrate targeting and, therefore, as noted above, can result in the unwanted alteration of the functions of proteins not related to disease, with potentially toxic consequences [41,42,43].

Our strategy here is to modify rationally Hsp70 to increase its activity and specificity for a selected target substrate by grafting onto it a binding peptide designed to interact with a given epitope in the target substrate. By increasing the binding efficiency towards the target substrate, this strategy avoids the need for overexpression or modification of the ATPase domain by increasing the efficiency towards the target substrate. We have previously shown that this approach can be used in vitro to alter Hsp70 to target specifically the amyloidogenic regions of α-synuclein, a disordered protein associated with Parkinson’s disease, resulting in a reduction of aggregation even at low molar ratios [44]. 

In this study, we targeted human islet amyloid polypeptide (hIAPP) since this peptide is the major component of the amyloid deposits found in patients with non-insulin dependent (type II) diabetes mellitus [45,46]. hIAPP is thought to play a significant role in the progressive loss of RIN-m5F β-cells, as hIAPP oligomers may interact with the outer membrane surface of these cells [45,46], and it is also known that hIAPP disrupts synthetic lipid vesicles [47]. Because of its association with this disease, substantial efforts have been devoted to finding ways of modulating the aggregation-dependent toxicity of hIAPP using small molecules [48,49,50]. The toxicity of hIAPP aggregates has been reduced by using antibodies that bind hIAPP oligomers, leading to a recovery of the toxic effects in neuroblastoma cells [51]. 

Our choice of targeting Hsp70 among other possible molecular chaperones was prompted by its known association with hIAPP-mediated pathology [52,53]. We provide a proof-of-principle of our approach by evaluating the activity of our designed Hsp70 variant in recovering the viability of a mammalian cell culture system. Specifically, we use the insulinoma cell line (RIN-m5F), as it is an established cellular model for studying the molecular mechanism of hIAPP-related toxicity [54]. In this system, aggregated hIAPP hinders cell viability by reducing proliferation rate and causing cell death by interacting with outer membranes [55,56]. 

## 2. Results

### 2.1. Rational Design of an Hsp70 Variant against hIAPP

**Figure 1:**
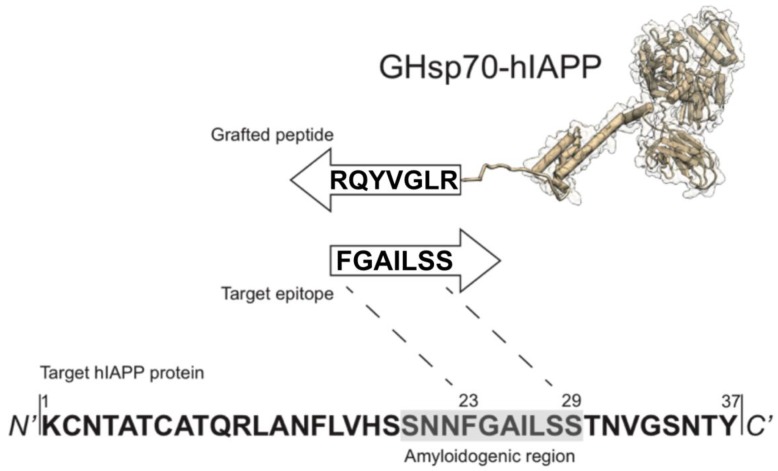
Schematic representation of the rational design strategy for generating a heat shock protein 70 kDa (Hsp70) variant specifically targeting the most amyloidogenic region of human islet amyloid polypeptide (hIAPP). The amino-acid sequence (RLGVYQR) was grafted at the C-terminal end of Hsp70, resulting in the grafted heat shock protein 70 kDa-human islet amyloid polypeptide (GHsp70-hIAPP) variant. The complementary peptide sequence targets the residues 23–29 in the amyloidogenic region of hIAPP [46,58,59].

In this work we rationally designed an Hsp70 variant with improved activity against hIAPP aggregation. The rational design procedure consists of two steps. First, we used a recently developed method [57] to construct the sequence of a complementary 8-residue peptide (RLGVYQR), targeting an 8-residue epitope (FGAILSS, Figure 1) on the hIAPP sequence in the vicinity of the region of residues 20–29, which is consistently recognised as amyloidogenic as it adopts a β-sheet structure upon amyloid fibril formation [46,58,59]. Second, we grafted this peptide on to Hsp70 by appending it to the C-terminus, thus generating a grafted variant, the grafted heat shock protein 70 kDa-human islet amyloid polypeptide (GHsp70-hIAPP) (Figure 1). The grafted peptide on GHsp70-hIAPP is expected to specifically bind its target epitope on hIAPP via complementary hydrogen bonding [44,57]. As controls, we used Hsp70 wild type (WT), and GHsp70-Aβ, which was designed with the same strategy of GHsp70-hIAPP, but carries a grafted complementary peptide designed to bind the 42-residue form of the Alzheimer’s amyloid β peptide (Aβ42) [44,57].

### 2.2. The Designed GHsp70-hIAPP Variant Increases Pancreatic Islet β-Cell Viability in Cell Culture Experiments

The viability of RIN-m5F cells was used to compare the effects of Hsp70 WT and the designed variant GHsp70-hIAPP on the cytotoxicity of hIAPP. A tetrazolium salt reduction assay (3-(4,5-dimethylthiazol-2-yl)-2,5-diphenyltetrazolium bromide) (MTT) was chosen since this assay is widely used in the literature to assess hIAPP toxicity in RIN-m5F cells [55,56].

**Figure 2:**
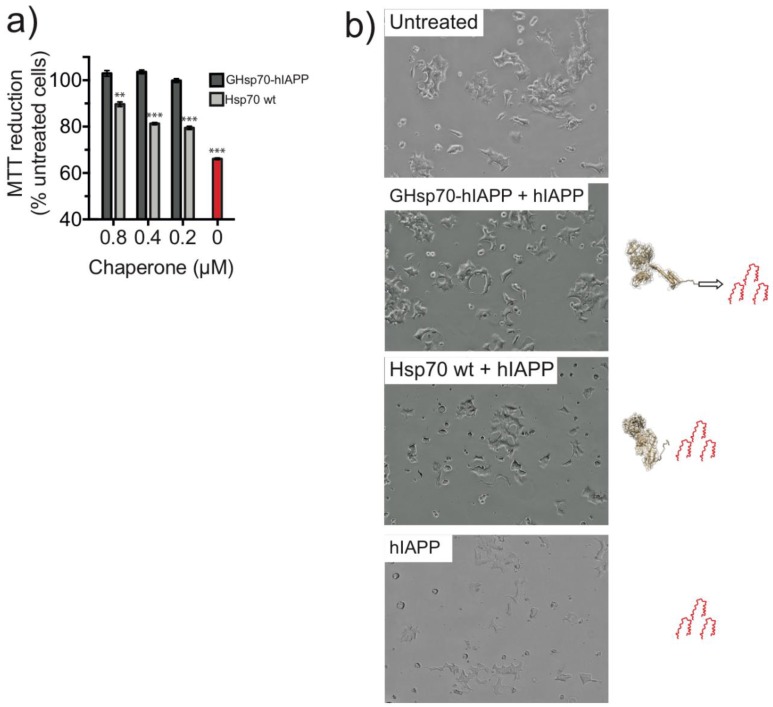
Treatment with the designed GHsp70-hIAPP variant increases the viability of cultured pancreatic islet β-cell*. *(**a**) 3-(4,5-Dimethyl-2-thiazolyl)-2,5-diphenyl-2*H*-tetrazolium bromide (MTT) reduction of cells incubated with 10 μM hIAPP at increasing molar concentrations of molecular chaperones (0.2, 0.4, 0.8 μM): GHsp70-hIAPP and 10 μM hIAPP (dark grey bars), Hsp70 WT and 10 μM hIAPP (light grey bars), and 10 μM hIAPP alone (red bar). Untreated cells were a positive control to assess the maximum MTT reduction, and cells lysed with Triton X-100 were used as a negative control to assess the minimum MTT reduction (~3 ± 0.5% untreated cells). Mean values were compared to data for untreated control cells using a Student’s *t*-test. ** *p* <0.01 and *** *p* <0.001; (**b**) Light microscopy images of RIN-m5F cells left untreated, or incubated with hIAPP in the presence of GHsp70-hIAPP or Hsp70 wild type (WT) (both at 0.8 μM), or incubated with hIAPP alone. Images are representative of cells from five replicate wells in a single experiment; the magnification is 15×.

We first checked that cell viability was significantly compromised in the presence of 10 μM hIAPP. We observed a reduction to a level (66 ± 1%), consistent with previous reports [55,56] (Figure 2a, red bar). In the presence of hIAPP, the addition of the GHsp70-hIAPP variant to the media for 24 h completely recovered the ability of RIN-m5F cells to reduce MTT to a level comparable to that of untreated cells (i.e., in the absence of hIAPP, black bar) for each molar concentration tested (Figure 2a). By contrast, the addition of Hsp70 WT resulted in only a partial recovery of the ability of treated RIN-m5F cells to reduce MTT. This effect was significantly lower compared to that of the GHsp70-hIAPP variant (at 0.8 μM, 89 ± 1% and 102 ± 1%, respectively, *p* < 0.01; at 0.4 μM 81 ± 1% and 103 ± 1%, respectively, *p* < 0.001; at 0.2 μM 79 ± 1% and 100 ± 1%, respectively, *p* < 0.001; Figure 2a). 

These findings are significant since they indicate that the action of the GHsp70-hIAPP variant takes place via a combination of a generic activity of Hsp70 and a specific activity of this molecular chaperone through the additional engineered interaction with hIAPP that we designed [44,57]. We then performed further control experiments using the Hsp70 variants alone in the presence of cells, and for both the wild type and the engineered variants, no significant changes in the ability of RIN-mF5 cells to reduce MTT compared to untreated cells were observed (Figure S1). Conversely, as expected, the presence of 10 μM hIAPP significantly hinders the ability of the cells to reduce MTT (Figure S2).

### 2.3. The Designed GHsp70-Aβ Variant Does Not Increase Pancreatic Islet β-Cell Viability

White-light images of RIN-mF5 cells exposed to hIAPP for 24 h displayed a marked difference in cell morphology (Figure 2b). hIAPP-treated cells displayed a less spreaded morphology and appeared less confluent compared to the untreated cells, indicating a reduction in the proliferation of these cells. These observations are consistent with previous studies that have reported a reduction in the proliferation of RIN-m5F cells via cell death processes induced by hIAPP exposure [54,60]. By contrast, cells treated with hIAPP in the presence of GHsp70-hIAPP exhibited a flattened and spreaded morphology highly similar to that of untreated cells (Figure 2b). Cells treated with hIAPP in the presence of Hsp70 WT displayed an intermediate growth morphology, somewhere between untreated and hIAPP-treated cells (Figure 2b). Taken together, the growth morphologies observed for each treatment appear commensurate with MTT reduction assays (Figure 2a). 

**Figure 3:**
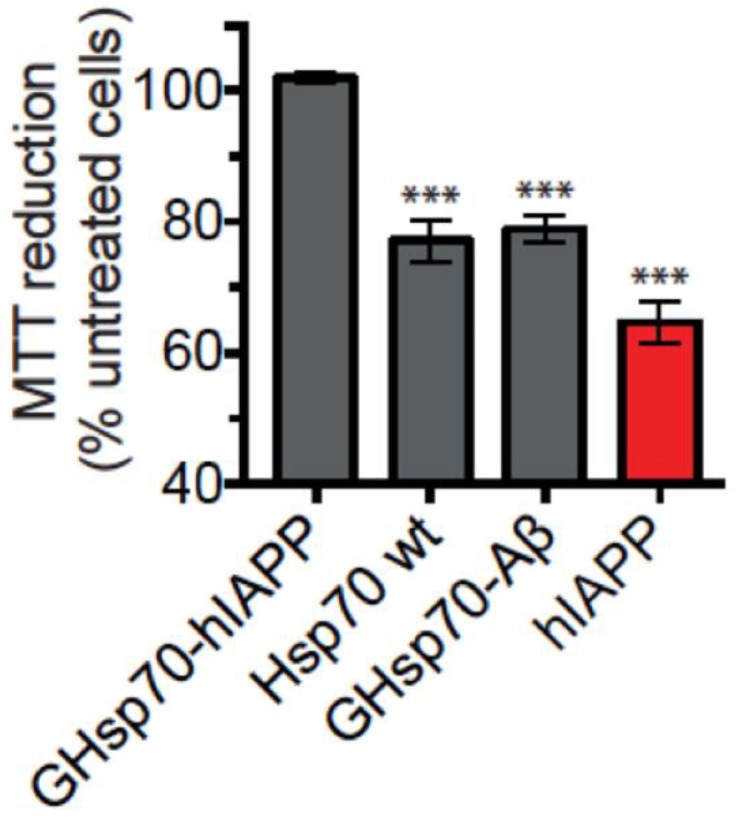
The designed GHsp70-hIAPP variant is specific in increasing the viability of cultured pancreatic islet β-cell. MTT reduction of cells incubated with hIAPP (10 µM) either alone (red bar) or with GHsp70-hIAPP (0.2 µM), Hsp70 WT (0.2 µM), and grafted heat shock protein 70 kDa- Alzheimer’s amyloid β peptide (Aβ42) (GHsp70-Aβ) (0.2 µM). The data shown are the mean ± SEM (*n *= 5) and are representative of two different experiments conducted on separate days. Mean values were compared to data for untreated control cells using a Student’s *t*-test. *** *p* < 0.001 are indicated by single, double, and triple asterisks, respectively.

We next used the GHsp70-Aβ variant as a control since this variant contains a C-terminal grafted extension of equal length and similar hydrophobicity and charge to the variant we grafted onto GHsp70-hIAPP. This is, therefore, a rather stringent test, as GHsp70-Aβ could be expected to enhance non-specific binding towards hIAPP aggregates compared to the Hsp70 WT, since its grafted peptide endows it with an enhanced inhibitory activity on the in vitro aggregation of Aβ42 [44]. In support of the specificity of the design procedure that we used in this work, we found that the MTT reduction activity of cells treated with hIAPP and GHsp70-Aβ is indistinguishable from that of cells treated with hIAPP and Hsp70 WT, which are both significantly lower than the activity of cells treated with hIAPP and Hsp70 GHsp70-hIAPP (Figure 3). 

### 2.4. The Designed GHsp70-hIAPP Variant Binds hIAPP with Higher Affinity than Wild-Type Hsp70

**Figure 4:**
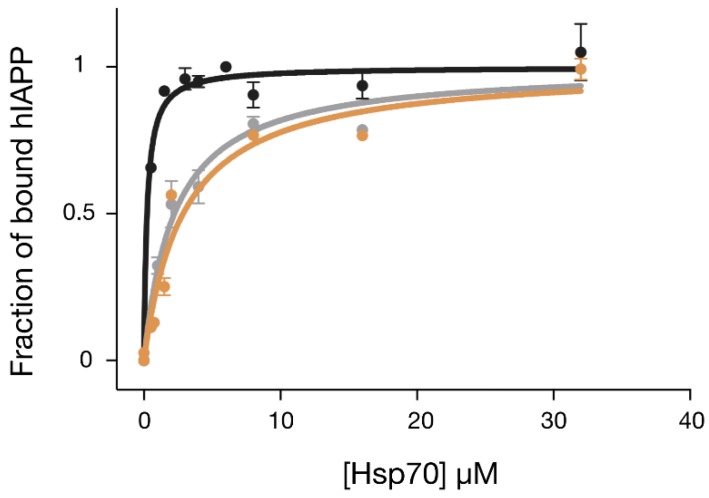
The designed variant GHsp70-hIAPP binds hIAPP better than the Hsp70 WT and GHsp70-Aβ variants. Fluorescence titration experiments for monitoring the binding of GHsp70-hIAPP (black), Hsp70 WT (grey), and GHsp70-Aβ (yellow) to 0.15 µM FAM-hIAPP. Data are reported as a fraction of bound hIAPP. Each point is the average of three independent measurements and the error bars are the standard deviations (SD). The continuous lines represent the best fits of the data to a single binding site model.

In order to understand whether the results from the cellular experiments could be explained by a stronger direct binding of GHsp70-hIAPP with hIAPP, we determined the affinity of this interaction and compared it to the corresponding affinities of Hsp70 WT and GHsp70-Aβ. To do so, we performed a fluorescence titration assay using a variant of hIAPP, which was N-terminally labelled with the fluorophore carboxyfluorescein, also known as FAM (Figure 4). In particular, we monitored the formation of the complex Hsp70:FAM-hIAPP by titrating increasing quantities of the molecular chaperone variants into solutions containing FAM-hIAPP and following the increase of fluorescence intensity of the FAM moiety. Binding data were all fitted with a single-binding-site model, as this simple model was in reasonable agreement with the data points in the concentration range explored and for the grafted variant. We found that Hsp70 WT was able to bind to hIAPP with a *K_d_* of 2.2 ± 0.3 μM. As expected, the designed variant GHsp70-hIAPP’s binding affinity was ten-fold higher (*K_d_*= 0.2 ± 0.06 μM), while the affinity of the control variant GHsp70-Aβ (*K_d_* = 2.8 ± 0.5 μM) was effectively unchanged with respect to that of Hsp70 WT.

As a further validation, we performed an ELISA-based binding assay (Figure S3). This analysis confirms, at least in a qualitative manner, that the designed variant GHsp70-hIAPP binds hIAPP better than Hsp70 WT and the control variant GHsp70-Aβ. Overall, consistently with the cellular assay, these in vitro results show that GHsp70-hIAPP has the highest binding affinity for hIAPP among all the Hsp70 variants under investigation.

### 2.5. The Designed GHsp70-hIAPP Variant Inhibits hIAPP Aggregation Better Than Wild-Type Hsp70 In Vitro

**Figure 5:**
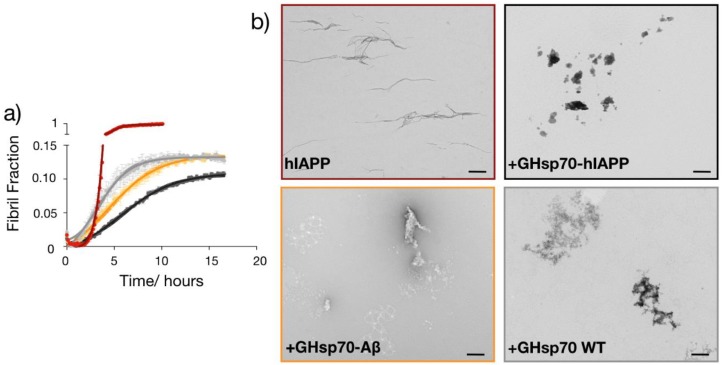
The designed variant GHsp70-hIAPP inhibits the aggregation of hIAPP more than the Hsp70 WT and GHsp70-Aβ variants. (**a**) Thioflavin-T (ThT) fluorescence assay of 10 µM hIAPP alone (red) or in the presence of 0.8 µM GHsp70-hIAPP (black), Hsp70 WT (grey) or GHsp70-Aβ (yellow); the data shown are mean values of three replicates and the error bars are the standard deviations (SD); (**b**) TEM images at the plateau of the aggregation reaction (15 h); the scale bar is equal to 500 nm.

Given the enhanced binding affinity of GHsp70-hIAPP for hIAPP, we then assessed whether this Hsp70 variant was also more effective in directly inhibiting its aggregation in vitro. To do so, we monitored the formation of amyloid fibrils in 10 μM solutions of hIAPP alone or in the presence of the three Hsp70 variants by means of thioflavin-T (ThT) aggregation assays in vitro (Figure 5a). In order to have a direct comparison with the cell toxicity assays, we performed the aggregations in the same medium used to test cell toxicity. We found that hIAPP alone forms fibrils within approximately 5 h under our experimental conditions, in agreement with previous studies [61,62]. We also observed that all the three Hsp70 variants have a very strong anti-aggregation activity, significantly reducing the amount of ThT-positive species formed during the aggregation of hIAPP (Figure 5a). In particular, GHsp70-hIAPP was the most active variant in doing so, in agreement with our other results. 

These aggregation experiments suggest that the reduction in hIAPP cell toxicity observed in the presence of the Hsp70 variants can be achieved via their anti-aggregation activity. As previous studies demonstrated that soluble oligomers formed during the aggregation of hIAPP are the most toxic species for RIN-m5F cells [63], we can speculate that Hsp70 is able to inhibit to some extent the formation of these toxic species. Interestingly, we noticed that the aggregation kinetics in the presence of Hsp70 are significantly different from that of hIAPP alone. In particular, this molecular chaperone seems to first accelerate the aggregation of hIAPP before suppressing it. This behaviour may indicate that Hsp70 can change the pathway of aggregation of hIAPP towards the formation of aggregates with different morphologies and toxicity. 

To obtain more insights into the specific mechanism of the Hsp70-mediated inhibition of hIAPP aggregation, we performed transmission electron microscopy for the same mixtures (i.e., 10 μM hIAPP alone or in the presence of the different Hsp70 variants) at the plateau of aggregation (15 h). We found that the morphology of the aggregates was significantly changed in the presence of all Hsp70 variants (Figure 5b), indicating that they are able to change the aggregation pathway of hIAPP.

As a control, we performed the same analysis at the beginning of the aggregation reaction of hIAPP alone, and after 15 h of a reaction containing the Hsp70 variants alone (Figure S4), finding no or very small aggregated structures.

## 3. Discussion

We have described a rational design strategy to generate a molecular chaperone variant targeting an epitope within the amyloidogenic region of hIAPP. We have applied this strategy to Hsp70 and showed that it resulted in a variant with an enhanced ability to inhibit the hIAPP-induced toxicity in cultured RIN-m5F pancreatic islet β-cells. We have also shown that this beneficial effect results from an increased anti-aggregation activity of the Hsp70 variant, which diverts the aggregation pathway of hIAPP towards the formation of non-toxic aggregates. 

The therapeutic potential of molecular chaperones is a field in its infancy. As a consequence, there are still concerns regarding the use of these molecules as therapeutic agents for protein misfolding diseases. These concerns include whether therapeutic molecular chaperones would effectively engage their targets and whether their administration would result in pro-inflammatory responses [9,10,11,12,13]. In the case of hIAPP-associated toxicity that we studied here, to facilitate the translation in vivo of the approach presented here, it will be interesting to repeat the present study with an extracellular molecular chaperone such as, for example, clusterin, for which target engagement should be more straightforward.

Although further methodological improvements are needed, our results provide support for the concept that engineered molecular chaperones could provide effective tools in biomedical and biotechnological applications related to protein misfolding and aggregation.

## 4. Materials and Methods

### 4.1. Hsp70 Variant Constructs

The different complementary peptides were grafted at the C-terminal end of human Hsp70 (human Hsp70 1A, GenBank entry NP005336) by means of mutagenic polymerase chain reaction (PCR) with phosphorylated oligonucleotides. Recombinant wild type and designed N-hexa-His-tagged Hsp70 variants were overexpressed from the pET-28b vector (Merck KGaA, Darmstadt, Germany) in *E. coli* BL21(DE3) Gold Strain (Agilent Technologies, Santa Clara, CA, USA) and purified as previously described [44].

### 4.2. Preparation of hIAPP for Cell Viability Tests

hIAPP (AnaSpec, Fremont, CA, USA) was dissolved at 1 mg/mL in hexafluoroisopropanol (HFIP) and incubated over night at room temperature in order to dissolve preformed aggregates. The following day, the peptide was lyophilised and resuspended in the cell medium just prior the toxicity experiments. 

### 4.3. Cell Culture

All cell culture reagents were purchased from Sigma-Aldrich (Dorset, England) unless otherwise specified. Pancreatic insulinoma RINm5F β-cells (ATCC, CRL11605, Rockville, MD, USA) from Rattus norvegicus were cultured in Roswell Park Memorial Institute (RPMI) 1640 medium (32404, Gibco, Loughborough, England) supplemented with 10% (*v/v*) fetal bovine serum (F9665), 1 mM sodium pyruvate (11360070, Gibco, Loughborough, England), 4500 mg/L glucose (G8644), 2 mM Glutamax supplement (35050061, Gibco, Loughborough, England), 20 mM HEPES (4-(2-hydroxyethyl)-1-piperazineethanesulfonic acid) and maintained at 37 °C, 5% CO_2_, and 95% relative humidity in tissue culture flasks (Greiner Bio-One, Kremsmünster, Austria). Cultures were routinely split (1:2) at ~80% confluency by rinsing the cells with Dulbecco’s phosphate buffered saline (DPBS, 10 mm) without calcium or magnesium and released for sub-cultivation using 0.25% (*v/v*) trypsin-EDTA.

### 4.4. Cell Viability Tests

Cells at ~80% confluency were detached from culture using 0.25% trypsin-EDTA, rinsed, then plated at a density of 50,000 cells per well in 150 μL of medium in a 96-well plate (clear or white, Greiner Bio-One, Kremsmünster, Austria) for 24 h prior to the experiments with hIAPP or Hsp70 variants. Cell viability assays were conducted during passage number 15–26 [54]. hIAPP was suspended in the cellular media, without fetal bovine serum (FBS) or sodium pyruvate, at a concentration of 10 μM, [55,60,63,64] and pre-incubated for 1 h in the cell media at 37 °C in the absence of cells since in this condition, hIAPP was observed previously to exert the highest toxicity (Figure S2). 

### 4.5. MTT Reduction Assay

RIN-m5F cell viability was assessed using the 3-(4,5-Dimethyl-2-thiazolyl)-2,5-diphenyl-2*H*-tetrazolium bromide (MTT; M5655, Sigma Aldrich) tetrazolium salt reduction assay, in 96-well plates. The intensity of the formazan produced from enzyme cleavage of the tetrazolium salt by metabolically active cells is proportional to the number of viable cells. After the exposure to treatments for 24 h at 37 °C, cells were incubated in a 0.5 mg/mL MTT solution per well for 2 h at 37 °C and then in a cell lysis buffer (20% SDS, 50% *N*,*N*-dimethylformamide, pH 4.7). The plate was gently mixed for 60 min at 400 rpm before the absorbance was measured at 570 ± 10 nm using a plate-reader (BMG Labtech, Offenburg, Germany). Untreated cells and cells lysed with 9% (*v/v*) Triton X-100 were used as controls for the expected maximum and minimum MTT reduction, respectively. Data are expressed as the percentage of MTT reduction compared to an untreated cell control corrected for the absorbance of cell media containing MTT without cells. 

### 4.6. ELISA Binding Assay

The wells of the ELISA plates (ThermoFIsher Scientific, Haverhill, MA, USA) were coated with 2.5, 5, and 10 µM of hIAPP and blocked with 5% (*w/v*) BSA in PBS (BSA/PBS). Samples of 60-µL of the different Hsp70 variants at 7 µM of protein concentration were then incubated in the coated wells. Primary mouse monoclonal antibody against human Hsp70 (C92F3A-5, Abcam, Cambridge, UK) and secondary antibody conjugated with the fluorophore Alexa-488 (ThermoFIsher Scientific, Haverhill, MA, USA) were diluted 1:1000 in BSA/PBS and added to the wells. All incubations were performed at room temperature for 1 h under shaking and were followed by six consecutive washes with PBS/Tween 0.02%. Fluorescence measurements were performed using a CLARIOstar plate reader (BMG Labtech, Allmendgruen, Germany).

### 4.7. Fluorescence Titration Assay

Solutions containing 0.15 µM FAM-hIAPP (Phoenix Pharmaceuticals, Inc., Burlingame, CA, USA) and different concentrations (from 0 to 36 µM) of either Hsp70 WT, GHsp70-Aβ, or GHsp70-hIAPP in 50 mM Tris pH 7.4, 150 mM KCl, and 5 mM MgCl_2_ were incubated in darkness for 1 h at room temperature (RT). At the end of the incubation, samples were transferred into a low-binding, clear-bottomed half-area 96-well plate (Corning Inc., New York, NY, USA). Fluorescence intensities were then recorded at 520 nm by exciting the samples at 490 nm with a CLARIOstar plate reader (BMG Labtech, Allmendgruen, Germany). They were subtracted for the value at 0 µM of chaperone and fitted using a one-site binding model with the software Prims (GraphPad Software, La Jolla, CA, USA). Data are represented as fraction of bound hIAPP, by setting the fitted value of the fluorescence plateau to 1.

### 4.8. Protein Aggregation Assay

Aggregation solutions containing 10 µM monomeric hIAPP alone or in the presence of 0.8 µM Hsp70 WT, GHsp70-Aβ, or GHsp70-hIAPP in cell medium and 20 µM ThT were incubated in quiescent conditions at 37 °C. ThT fluorescence of the samples was monitored at 480 nm in low-binding, clear-bottomed half-area 96-well plates (Corning Inc., New York, NY, USA) upon excitation at 440 nm by means of a CLARIOstar plate reader (BMG Labtech, Ortenberg, Germany). Fluorescence data were then converted into relative fibril mass, assuming the fluorescence value of hIAPP at the plateau alone was equal to 1.

### 4.9. Transmission Electron Microscopy (TEM)

TEM images of hIAPP aggregates obtained in absence or presence of the chaperone variants were acquired using a Tecnai G2 80–200 kv transmission electron microscope (ThermoFIsher Scientific, Haverhill, MA, USA). Samples of 10 µL were applied to 400 mesh copper grids, washed twice with ddH_2_O, and negatively stained with 2% (*w/v*) uranyl acetate.

## Supplementary Materials

Supplementary materials can be found at http://www.mdpi.com/1422-0067/19/5/1443/s1.

## Abbreviations

Hsp70	Heat shock protein 70 kDa
GHsp70	Grafted Heat shock protein 70 kDa
hIAPP	Human islet amyloid polypeptide
MTT	3-(4,5-Dimethyl-2-thiazolyl)-2,5-diphenyl-2*H*-tetrazolium bromide
ThT	Thioflavin-T
