# Supplementary material for: A Rationally Designed Hsp70 Variant Rescues the Aggregation-Associated Toxicity of Human IAPP in Cultured Pancreatic Islet β-Cells
Source: Int J Mol Sci. 2018 May 12;19(5):1443. doi: 10.3390/ijms19051443 (PMC5983706; doi:10.3390/ijms19051443)
Supplement: Supplementary file 1 [file ijms-19-01443-s001.pdf]

SUPPLEMENTARY INFORMATION

A rationally designed Hsp70 variant rescues the aggregation-associated toxicity of human IAPP in cultured pancreatic islet β-cells

Marie Nicole Bongiovanni, Francesco Antonio Aprile*, Pietro Sormanni and Michele Vendruscolo*

Centre for Misfolding Diseases, Department of Chemistry, University of Cambridge, Cambridge, CB2 1EW, UK

Correspondence to: F. A. A. (faa25@cam.ac.uk) or M. V. (mv245@cam.ac.uk)

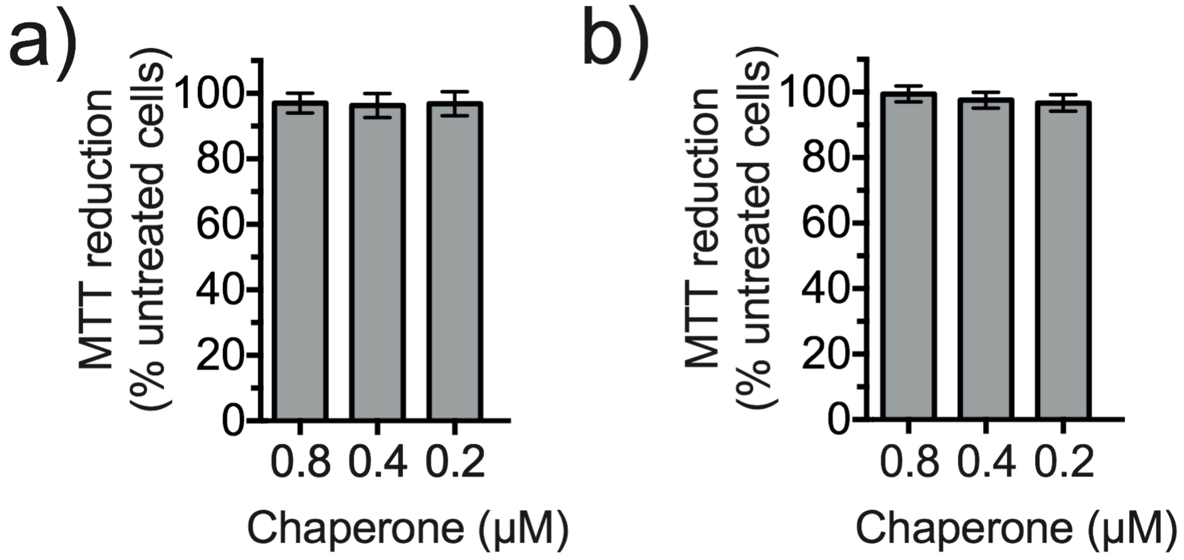

Figure S1. Viability of RIN-m5F cells in the presence of increasing concentrations of Hsp70 variants, but not of hIAPP, using the MTT reduction assay. Cells were incubated for 24 h with: **(a)** Hsp70 WT or **(b)** GHsp70-hIAPP at the molar concentrations used in **Figure 2a**. Data are the mean ± SEM (*n* = 5) and are representative of two different experiments conducted on separate days. Cells lysed with Triton X-100 were used as a negative control to assess the minimum MTT reduction (~2 ± 0.5 % of untreated cells).

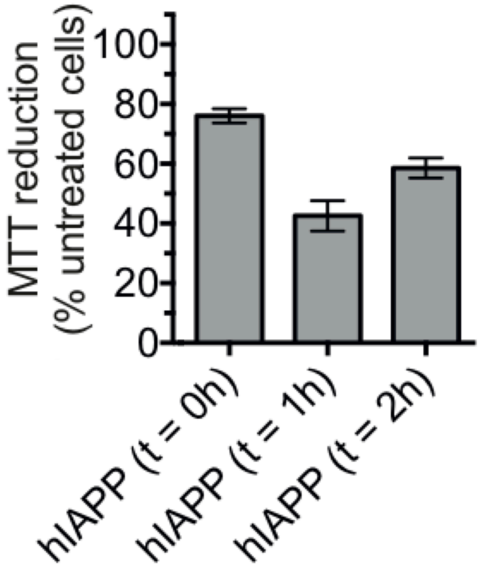

Figure S2. Toxicity of hIAPP on RIN-m5F cells using the MTT reduction assay. Cells were incubated for 24 h with hIAPP suspended at 10 μM in cell media. The media was added to the cells immediately (t = 0 h) or after pre-incubation at 37 °C at quiescent conditions for 1 hour (t = 1h) or 2 hours (t = 2h). Data are the mean ± SEM (*n* = 5) and are representative of two different experiments conducted on separate days. Cells lysed with Triton X-100 were used as a negative control to assess the minimum MTT reduction (~3 ± 0.5 % of untreated cells).

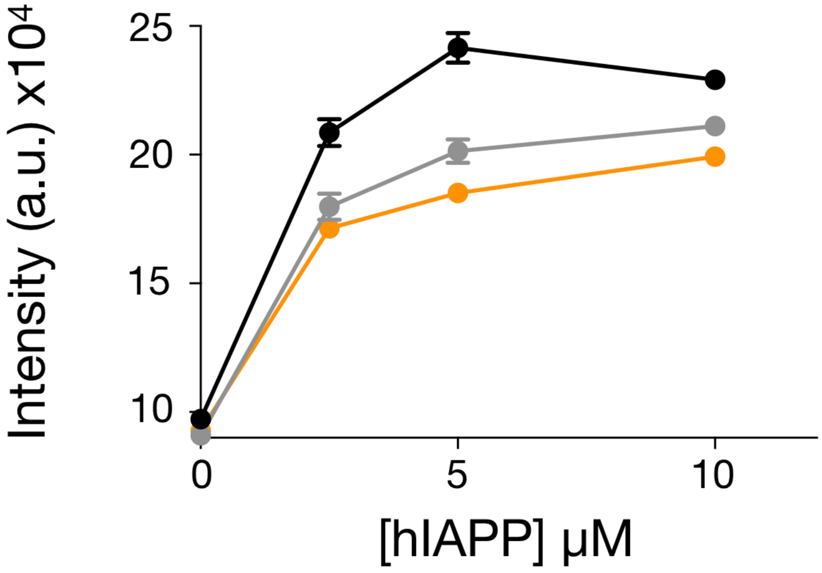

Figure S3. ELISA binding assay of hIAPP (2.5, 5, 10 µM) with GHsp70-hIAPP (black), Hsp70 WT (grey) and GHsp70-Aβ (yellow) variants.

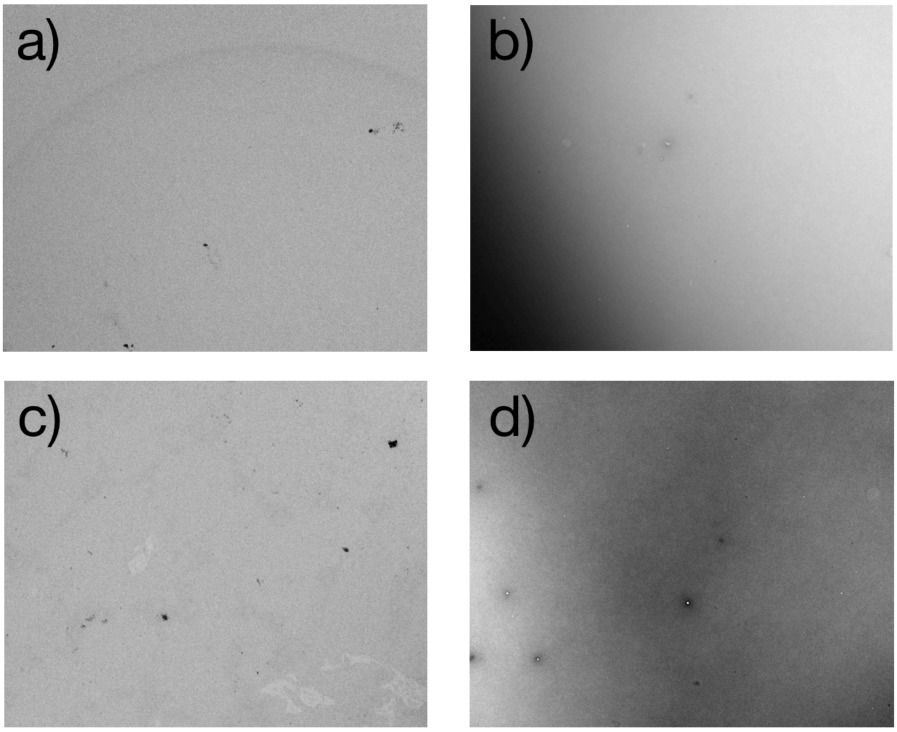

Figure S4. **(a)** TEM image of a solution containing 10 μM hIAPP alone at the beginning of the aggregation reaction. **(b-d)** TEM images of solutions containing 0.8 μM Hsp70 WT **(b)**, GHsp70-Aβ **(c)**, or GHsp70-hIAPP **(d)** in the absence of hIAPP after 15 hours of incubation at 37 °C.
